# A Cotton Rope in the Colon: A Rare Cause of Chronic Abdominal Pain

**DOI:** 10.14309/crj.0000000000001251

**Published:** 2024-01-16

**Authors:** Imran Ali Syed, Kashif Rafi, Hafiz Muhammad Wasif Khan, Ahmad Karim Malik, Usman Iqbal Aujla

**Affiliations:** 1 Gastroenterology and Hepatology Department, Pakistan Kidney and Liver Institute & Research Center, DHA Phase VI, Lahore, Pakistan

**Keywords:** gossypiboma, abdominal pain, diathermic needle knife, endoscopic innovation

## Abstract

Retained surgical sponge is a relatively rare medical condition with potential serious medicolegal implications. The term “gossypiboma” is frequently used to describe this condition. We present a case of a 40-cm-long retained surgical sponge in a 43-year-old woman who presented with unexplained chronic abdominal pain for several years. She had a history of open cholecystectomy, hepaticojejunostomy, and enteroenterostomy. Computed tomography scan revealed a large cotton sponge anchored within the large bowel. Surgical exploration is usually required for the retrieval of gossypiboma. However, it was successfully removed endoscopically using a diathermic needle knife with no immediate complications. The patient was discharged after 48 hours with marked improvement in her abdominal pain. This case emphasizes the emerging role of novel endoscopic interventions, resulting in excellent clinical outcomes, avoiding major surgical interventions, and providing cost-effective benefits.

## INTRODUCTION

The term “gossypiboma” is used to describe retained, nonabsorbable surgical material usually composed of cotton, which is left inside the body after surgery. The word “gossypiboma” originated from *gossypium* (Latin word meaning cotton) and “boma” (Swahili word meaning place of concealment).^1^ Another term used for this medical condition is “textiloma,” which is derived from a combination of the Latin word *textilis* (weave) and the Greek word *oma* (swelling).^2^ Its incidence varies between 1 of 300–1,000 of all surgeries, and for intra-abdominal surgeries, its reported incidence is 1 of 1,000–1,500.^3^ In developing nations, its incidence might be underreported because of medicolegal implications and relatively less-established governance structures.

The spectrum of presentation ranges from incidental findings in an asymptomatic patient to variable presentations. Presentation can be acute or chronic. Birolini et al^4^ analyzed 4,547 retained foreign bodies containing 90% textiloma. Retention was identified within 2 months in 42%, between 2 and 12 months in 36%, between 1 and 5 years in 14%, and beyond 5 years in 8% cases. Symptomatic patients may present with abscess formation, fistula, peritonitis, adhesions, and migration into the gastrointestinal tract, causing obstruction. The most frequent site for gossypiboma is the abdominal cavity (56%), followed by the pelvic cavity (18%) and thoracic cavity (11%).^5^

We present a case of unexplained abdominal pain because of a retained surgical sponge in the large bowel for more than 4 years and the unique utility of an endoscopic intervention for its successful retrieval.

## CASE REPORT

A 43-year-old woman presented with a few weeks' history of jaundice and chronic abdominal pain for the last 4 years. The abdominal pain was colicky and intermittent with no radiation or aggravating factors. The pain was mild to moderate intensity and was responsive to antispasmodics and analgesics. She often experienced constipation but denied any history of vomiting, abnormal eating habits, hematochezia, or weight loss.

Her medical history revealed a laparoscopic cholecystectomy performed 4 years ago at a local hospital because of chronic cholecystitis. However, the cholecystectomy was complicated by the bile duct injury, resulting in obstructive jaundice that required hepaticojejunostomy. The surgery was further complicated by intra-abdominal collections and adhesions, which necessitated a second laparotomy leading to enteroenterostomy.

**Figure 1.**
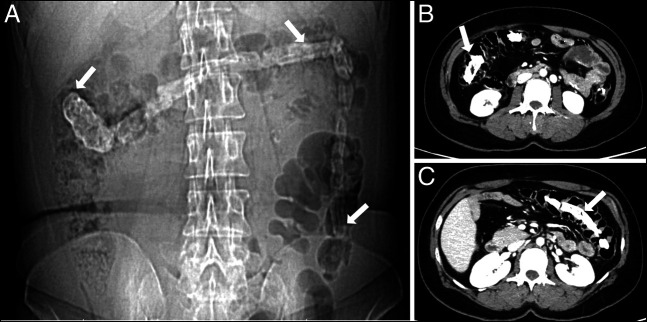
Radiological appearance of gossypiboma. (A) Retained surgical sponge extending from right colon into the transverse colon and up to descending colon (white arrows). (B, C) Abdominal computed tomography scan (axial section) showing gossypiboma within the large bowel loops (white arrows).

**Figure 2.**
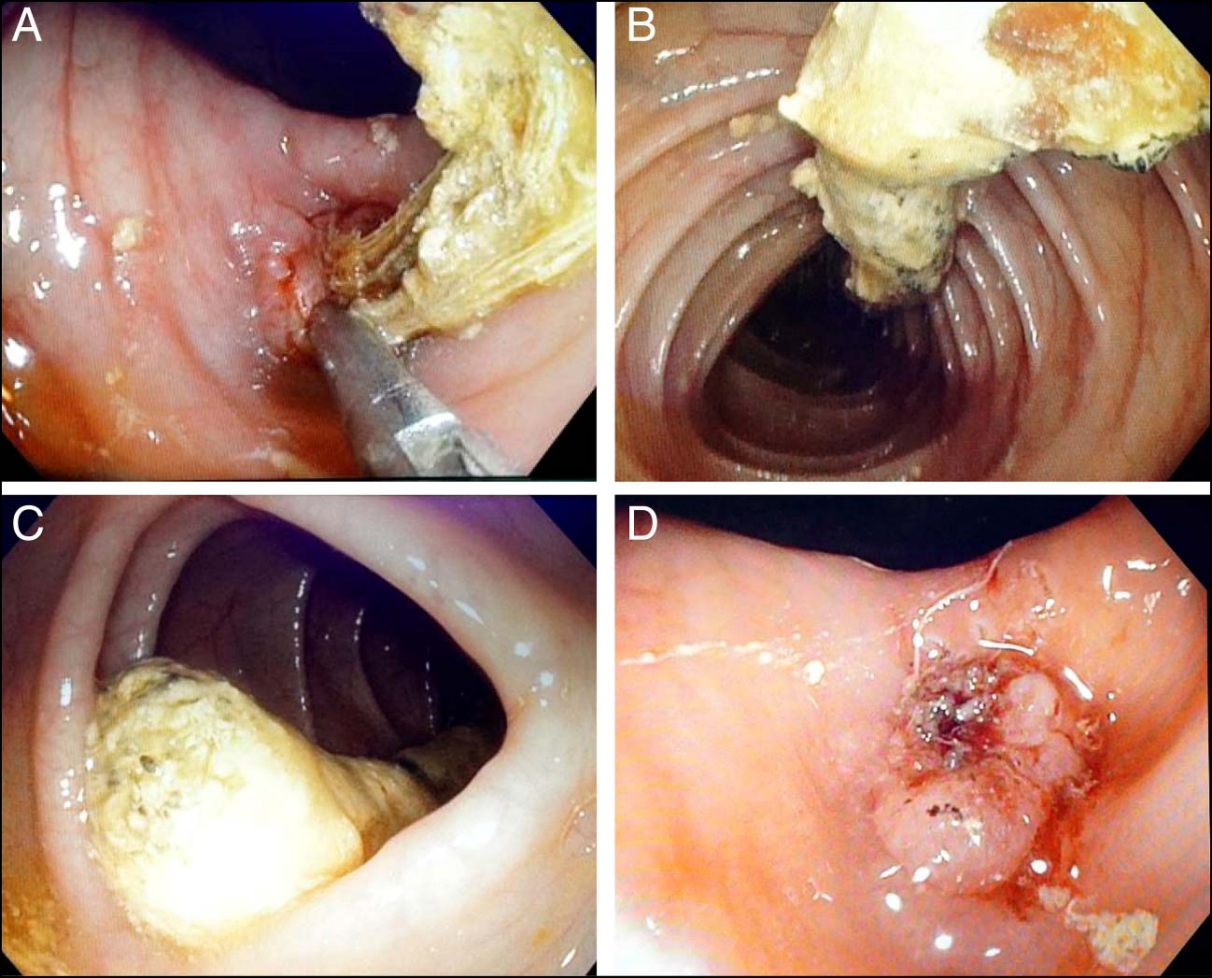
Endoscopic view of gossypiboma. (A) Proximal end of retained surgical sponge anchored into the ascending colon mucosa. (B, C) Gossypiboma extending into the transverse colon and up to descending colon. (D) Anchoring point of gossypiboma after its successful resection and retrieval showing nodularity and erythema.

**Figure 3.**
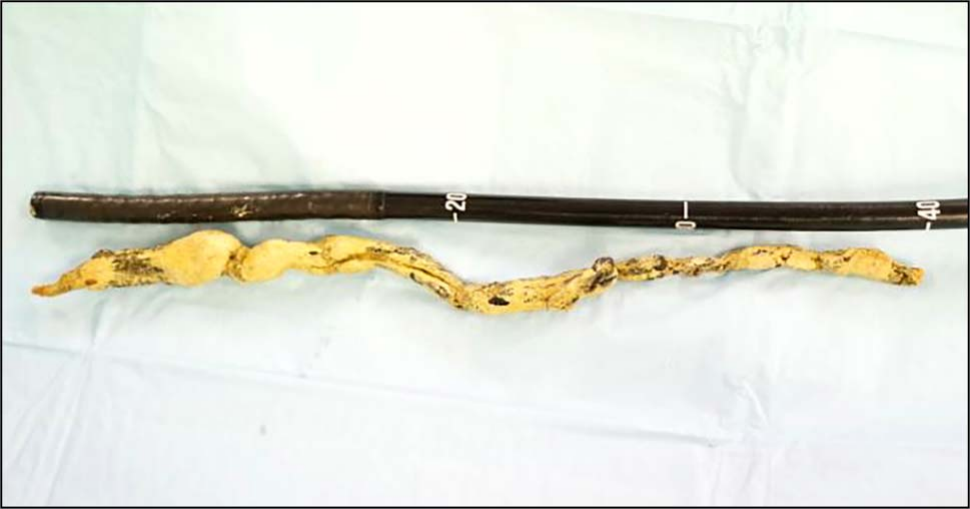
40-cm-long surgical sponge retrieved successfully.

**Figure 4.**
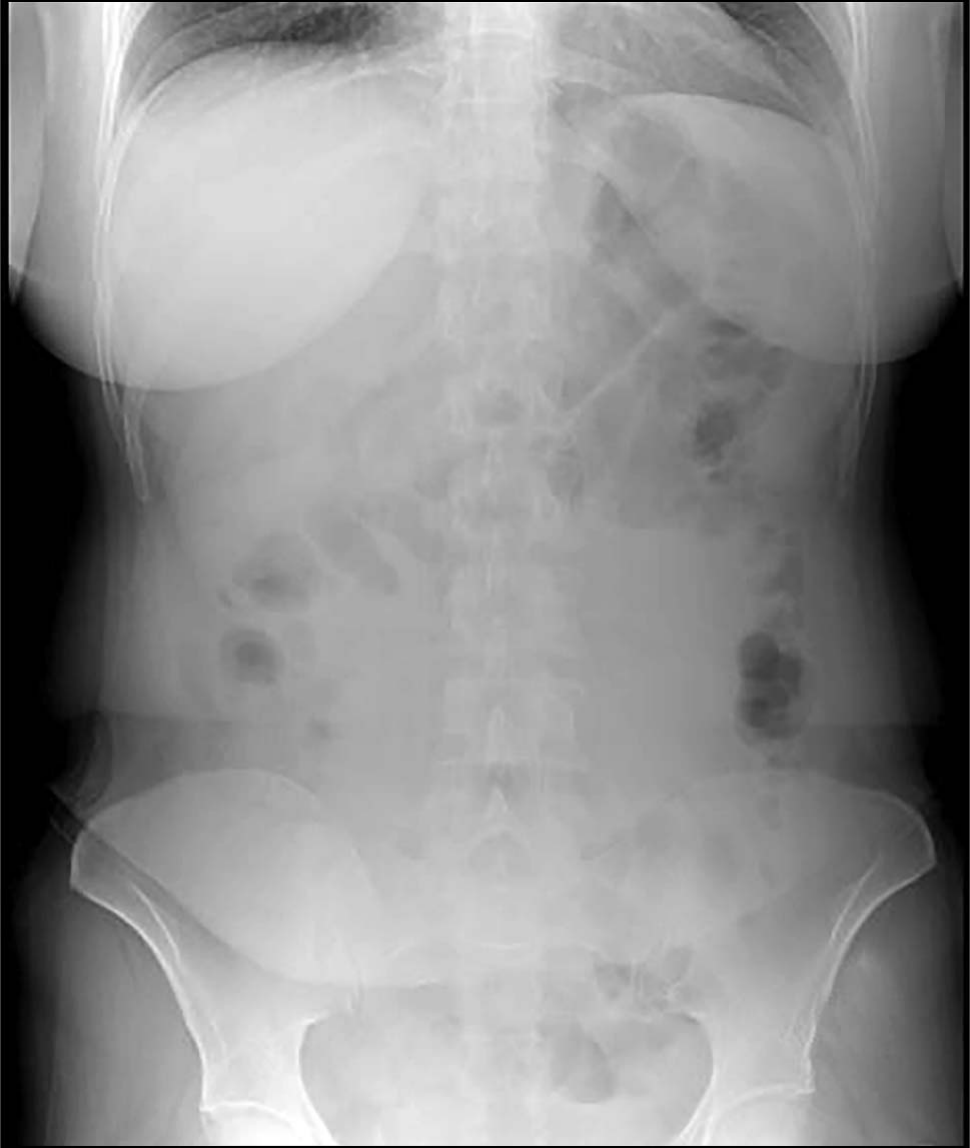
X-ray abdomen erect showing no signs of pneumoperitoneum after endoscopic removal of gossypiboma.

She was referred to our unit for evaluation of unexplained abdominal pain and jaundice. Her laboratory investigations were unremarkable except for cholestatic liver function tests (total bilirubin = 4.53 mg/dL with a predominant direct component, alkaline phosphatase = 447 U/L, and gamma-glutamyl transferase = 938 U/L). A contrast-enhanced abdominal computed tomography (CT) scan revealed hepaticojejunostomy site stricture with resultant intrahepatic biliary duct dilatation. The CT scan further showed a 27-cm-long tubular calcified foreign body within the large bowel, extending from the hepatic flexure to the mid-descending colon with few linear internal lucencies, raising suspicion of a misplaced stent with encrustation or a chronically retained sponge (Figure 1). Subsequently, a colonoscopy was performed, which showed a foreign body with its proximal end embedded in the ascending colon (Figure 2) and distal end extending up to the descending colon (Figure 2). The surrounding mucosa at the proximal attachment site was nodular with a mild local inflammatory response (Figure 2). The visible fibers of the retained sponge were cut and safely released from the point of origin using a diathermic needle knife. The foreign body was grasped from its distal end with a snare and retrieved along the colonoscope, showing a 40-cm-long and 5-cm-wide retained sponge (Figure 3). The patient was then transferred back to the inpatient services without immediate complications. A postcolonoscopy abdominal x-ray revealed no evidence of bowel perforation or obstruction (Figure 4). After 48 hours of observation and significant improvement in her abdominal pain, the patient was discharged and referred to surgeons for a redo hepaticojejunostomy to address the jaundice. The patient remained pain-free at a 6-month follow-up.

## DISCUSSION

Gossypiboma is a relatively rare condition with serious medicolegal implications.^6^ Gossypibomas are considered “never events” in healthcare, as clearly stated in patient safety guidelines by the National Quality Forum of the United States of America and the Health Department of the United Kingdom.^5,7^

Pathologically, gossypiboma causes foreign-body reactions of 2 types: either abscess formation resulting from an exudative reaction, leading to early detection, or an aseptic fibrinous reaction resulting in a sterile foreign-body granuloma with longer asymptomatic retention. The omentum and intestinal loops may surround the gossypiboma to encapsulate it, causing pressure necrosis of the intestinal wall and migration into the lumen.^8,9^ Intestinal penetration can manifest in any segment of the intestinal tract, with a higher incidence observed in the ileum and the colon.^10,11^

Radiography is the most commonly used modality for identifying radio-opaque marker tagged textiloma with a sensitivity >90%. Ultrasound could be favored as the primary imaging modality with reported sensitivity approaching 98%. CT is highly sensitive and specific in identifying gossypiboma, whereas magnetic resonance imaging has a reported sensitivity of around 100%.^12,13^

Surgery is the conventional method for retrieval when there is evidence of adhesion and/or partial displacement, whereas the utilization of minimally invasive approaches such as endoscopy may be feasible depending on the gossypiboma location and the availability of expertise. When there is transmural migration of gossypiboma in the digestive tract without evidence of perforation or obstruction, endoscopy might serve as a viable option for their extraction.^14–16^ In this particular case, endoscopic innovation avoided a major surgical intervention with the novel utilization of a diathermic needle knife during colonoscopy, resulting in the successful retrieval of the embedded surgical sponge. There was minimal self-limiting bleeding, which settled spontaneously without any immediate or delayed complications, including perforation and peritonitis.

To mitigate the risk, implementing precautions such as staff education, the use of marker-tagged sponges, or conducting perioperative sponge and material counts can effectively reduce the occurrence of gossypiboma.^17^ Adoption of innovative technologies (Electronic Computer Assisted Sponge Counting System and Radio-Frequency Identification sponges) may offer promising avenues for reducing such incidents.^18,19^

Postoperative patients manifesting unexplained symptoms and ambiguous radiological findings should raise suspicion for gossypiboma. This case highlights the evolution of innovative endoscopic interventions to avoid surgical exploration with related morbidity. Endoscopic interventions provide faster recovery, reduced hospital stay, and improved clinical outcomes with cost-effective benefits.

## DISCLOSURES

Author contributions: I. Syed: wrote the manuscript and reviewed the literature. K. Rafi: provided endoscopic images and described the findings. HM Wasif Khan: reviewed the literature and manuscript revision. AK Malik: literature review and manuscript revision. UI Aujla: manuscript writing, critical analysis, and final approval and is the article guarantor.

Financial disclosure: None to report.

Informed consent was obtained for this case report.
